# Physician preference for receiving machine learning predictive results: A cross-sectional multicentric study

**DOI:** 10.1371/journal.pone.0278397

**Published:** 2022-12-14

**Authors:** Roberta Moreira Wichmann, Thales Pardini Fagundes, Tiago Almeida de Oliveira, André Filipe de Moraes Batista, Alexandre Dias Porto Chiavegatto Filho

**Affiliations:** 1 School of Public Health, University of São Paulo, São Paulo, São Paulo, Brazil; 2 Brazilian Institute of Education, Development and Research – IDP, Economics Graduate Program, Brasilia, Federal District, Brazil; 3 Clinics Hospital of Ribeirão Preto of the University of São Paulo, Ribeirão Preto, São Paulo, Brazil; 4 State University of Paraiba, Campina Grande, Paraíba, Brazil; 5 Insper, Institute of Education and Research, São Paulo, São Paulo, Brazil; Business on Engineering and Technology S.A.C (BE Tech), PERU

## Abstract

Artificial intelligence (AI) algorithms are transforming several areas of the digital world and are increasingly being applied in healthcare. Mobile apps based on predictive machine learning models have the potential to improve health outcomes, but there is still no consensus on how to inform doctors about their results. The aim of this study was to investigate how healthcare professionals prefer to receive predictions generated by machine learning algorithms. A systematic search in MEDLINE, via PubMed, EMBASE and Web of Science was first performed. We developed a mobile app, RandomIA, to predict the occurrence of clinical outcomes, initially for COVID-19 and later expected to be expanded to other diseases. A questionnaire called System Usability Scale (SUS) was selected to assess the usability of the mobile app. A total of 69 doctors from the five regions of Brazil tested RandomIA and evaluated three different ways to visualize the predictions. For prognostic outcomes (mechanical ventilation, admission to an intensive care unit, and death), most doctors (62.9%) preferred a more complex visualization, represented by a bar graph with three categories (low, medium, and high probability) and a probability density graph for each outcome. For the diagnostic prediction of COVID-19, there was also a majority preference (65.4%) for the same option. Our results indicate that doctors could be more inclined to prefer receiving detailed results from predictive machine learning algorithms.

## Introduction

Doctors, nurses, physiotherapists, psychologists, among other healthcare professionals, face a massive amount of health information, and traditional ways of managing and evaluating information may not be enough for an efficient use of these resources [1]. A large number of information technologies (e-health applications) are already being used by organizations and individuals to make healthcare data applicable to patients [2].

Applications based on Artificial Intelligence (AI), specifically on Machine Learning (ML), have been on the rise in the last decade [3]. For example, machine learning-based diagnostic models have been developed to identify individuals with influenza [4]. A recent study carried out by US researchers validated a prediction model for Ebola patients, later deployed into a mobile app [5]. During the COVID-19 pandemic, geographic tracking applications were also developed to identify clusters of infected individuals and to improve collective decisions based on these results [6].

Some of the resistance from physicians in using e-health solutions may be related to the fear of new responsibilities [7]. Thus, interventions that require too many adaptations by the professionals may not be very effective. In addition, it has been suggested that presenting the result by asking the professional to make a specific decision (e.g. to intubate) or showing scores that are difficult to understand should be avoided [8]. In order to work directly with predictive algorithms, physicians will need skills usually not taught during medical training, such as performing and interpreting advanced calculations, database management, and programming [9]. For AI to have its potential fully implemented in healthcare, it is important that users are able to interpret the outputs of these predictions. Doctor-patient relationships can be negatively impacted if the professional does not know how to communicate uncertainties from a decision that was taken with the help of AI-based applications [10].

The predictive performance of a model may be insufficient to determine the success and popularity of a ML-based medical application among health professionals [11]. The presentation of the results, its usability and the amount of information provided can directly impact the use of the application. Equipping physicians with easy-to-use and accessible algorithms can help improve decisions especially in the face of a new disease scenario [12]. Regarding medical mobile applications, developers should also consider the usability, which is defined by The International Organization for Standardization as "the extent to which a product can be used by a defined group of users to achieve specific goals with effectiveness, efficiency and satisfaction in a given context of use” [13]. There are validated questionnaires for assessing the usability of applications in general, not necessarily medical, such as the System Usability Scale Task Completion (SUS) and the Suitability Assessment of Materials (SAM), which assess the complexity, integration, need for previous knowledge to use it, and presence of inconsistency of the applications, among other factors [14].

To our knowledge, there are still no tools available for usability assessment of ML-based eHealth applications. There are also no studies that investigate the level of technical details of these results that should be shown to healthcare professionals. The aim of this study is therefore to test how health professionals prefer to receive the results of ML-based prediction models and to contribute to the improvement of future technologies that use AI algorithms.

## Methods

A mobile application, RandomIA, was developed by the IACOV-BR (Artificial Intelligence for COVID-19 in Brazil) network to provide doctors with predictions of negative health outcomes. Patient data such as sex, age, vital signs, and complete blood count were used to develop the predictive algorithm (S1 Table). The study was approved by the Research Ethics Committee of the School of Public Health of the University of São Paulo (CAAE: 44298521.1.0000.5421).

### Study design

A search in EMBASE and MEDLINE via PubMed was conducted using the terms “mobile application”, “artificial intelligence” and “COVID-19” to identify studies that developed questionnaires for validating e-health applications, specifically regarding those employing machine learning algorithms. Then, a cross-sectional multicentric study was conducted from November 2021 to March 2022 by applying a questionnaire to physicians from the IACOV-BR network. Each professional had access to RandomIA, where they were asked to input clinical data from at least three patients affected by COVID-19. Three distinct types of results were presented with varying degrees of complexity. Finally, the physicians answered a questionnaire that asked to order the type of results they preferred. A group of 20 questions were selected (S2 Table) and incorporated into RandomIA. The first ten questions are part of a widely validated form, the System User’s Usability scale (SUS), which quantifies the interaction between users and an application. All participants accepted the informed consent form to evaluate the application.

### Population

The main inclusion criterion for the study was to be a physician in a hospital from the IACOV-BR network associated with the project. After selecting 18 hospitals to participate in the study, a convenience sampling of medical users was conducted and a minimum number of doctors was established to compose the group. This was determined based on a finite number of physicians from the IACOV-BR institutions (30,000) and the calculation of the sample size from a simple random sample [15]. The minimum sample size of physicians to represent this specific population was estimated at 68 sample units (n = 68), when considering a confidence level of 90% [16].

### RandomIA

**Fig 1:**
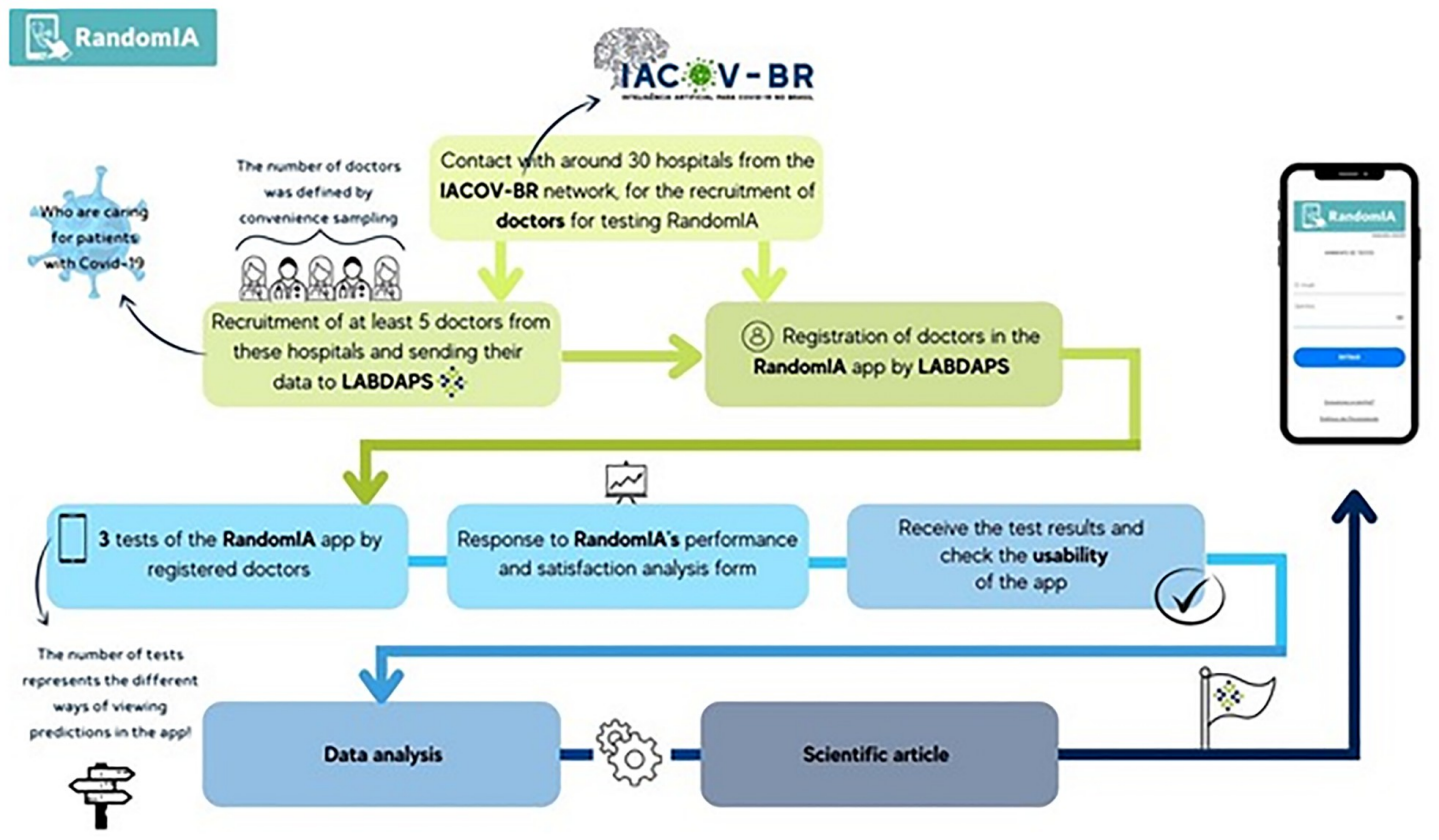
Flowchart of RandomIA assessment activities.

**Fig 2:**
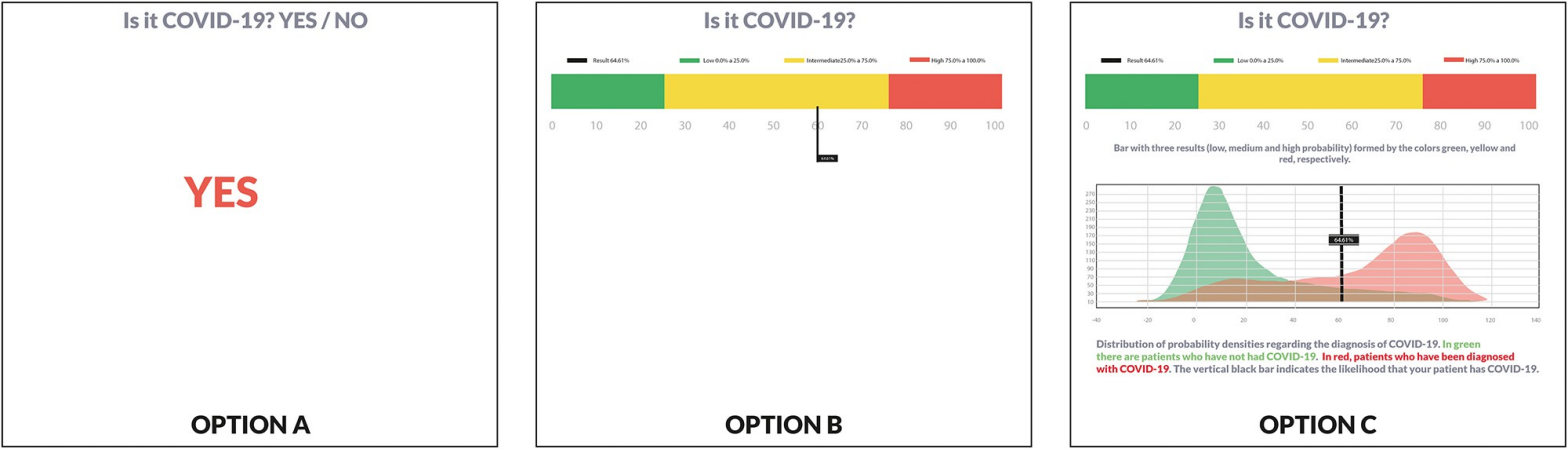
Predictive results shown by the RandomIA application according to three different levels of complexity.

RandomIA is a digital application that aims to use artificial intelligence algorithms to provide diagnostic and prognostic predictions for COVID-19 (Fig 1) The testing process by the physician began with the login to RandomIA via the random-ia.com URL and the filling in of the mandatory login and password fields, previously created and made available to each user by the research group. After logging into the application, the following options appeared on the screen: new prediction, history, learn more and evaluation survey. The user was then able to select “new prediction” to start filling the available fields with data from patients for which a RT-PCR exam was performed, regardless of the result (positive or negative). This process was performed three different times by each physician, using data from three patients to then visualize three different ways of receiving the prediction results (Fig 2).

### Statistical analysis

Descriptive statistics of the results were performed, in which numerical variables were presented as means and standard deviations, and categorical variables as absolute and relative frequencies. The variables originally distributed on a Likert scale are those which the response required participants to classify their preferences according to the following possibilities: “strongly disagree”, “disagree”, “neither agree nor disagree’’, “agree” and “strongly agree” on an equal distance scale [17]. For interpreting the results of the analysis, we considered that “strongly disagree” and “disagree” were low grades, that is, unfavorable to the statement of the item. On the other hand, the options “strongly agree” and “agree’’ represented the high grades of the Likert scale. Column graphs on the Likert scale were created using the R Likert package [18].

An ordinal multinomial regression analysis was also performed. Here we used the Akaike information criterion-based approach to determine if the data fitted as likely the model as a model that suggests no difference between the groups (nested models). At the same time, likelihood ratio tests were applied to the nested models to assess the hypothesis that the answers were parallel. This verification was performed via a chi-square test statistic χ2 for the likelihood ratio [19]. The probabilities were plotted in a line chart for comparison, using the net package from R, version 4.0.3. Factor Analysis and Principal Component Analysis aim to preserve the original variability of the data by summarizing variables correlated with each other into a smaller set of uncorrelated variables that gather the most information from the original set [20]. A scree-plot graphic approach was used [21], in order to assess whether there were differences between the groups in terms of sex, age, medical specialty, and region of the country.

## Results and discussion

A total of 69 physicians were recruited and asked to evaluate the options to visualize the result after testing the application at least three times.

Table 1 presents the descriptive results of the questionnaire. Variable Q1 (“I would use the app frequently”) had an average response of 3.36, slightly higher than the median of 3.0, indicating a higher proportion of physicians likely to use the app. On the other hand, variable Q2 (“I considered the application hard to use”), had an average of 1.87, demonstrating the ease of use of the application and corroborating the result of variable Q1. For variable Q3, (“I found the application easy to use”), 25% of the participants rated it up to 4 on the Likert scale, while the remaining 75% responded 4 or 5 on the same scale, suggesting an easy use of RandomIA. For variable Q4 (“I would need help from a person with technical knowledge to use the application”), an average of 1.38 was found, reinforcing the result obtained for variable Q3.

**Table 1:** Descriptive analysis for Likert scale variables.

Variable	Mean (SD)	Likert Scales					Percentiles				
		1	2	3	4	5	P_0_	P_25_	P_50_	P_75_	P_100_
Q1	3.36 (1.25)	10.1	14.5	24.6	30.4	20.3	1	3	4	4	5
Q2	1.87 (1.00)	42	39.1	13	1.4	4.3	1	1	2	2	5
Q3	4.22 (1.17)	2.9	11.6	7.2	17.4	60.9	1	4	5	5	5
Q4	1.38 (0.91)	78.3	14.5	2.9	0	4.3	1	1	1	1	5
Q5	3.99 (0.92)	0	5.8	24.6	34.8	34.8	2	3	4	5	5
Q6	2.35 (1.27)	33.3	26.1	20.3	13	7.2	1	1	2	3	5
Q7	4.46 (0.90)	0	7.2	5.8	20.3	66.7	2	4	5	5	5
Q8	1.74 (1.04)	55.1	27.5	8.7	5.8	2.9	1	1	1	2	5
Q9	3.77 (1.26)	8.7	7.2	18.8	29	36.2	1	3	4	5	5
Q10	1.43 (0.79)	71	18.8	5.8	4.3	0	1	1	1	2	4
Q11	2.71 (1.24)	23.2	18.8	27.5	24.6	5.8	1	2	3	4	5
Q13	2.96 (1.24)	15.9	21.7	21.7	31.9	8.7	1	2	3	4	5

*P50 50th percentile equals the Median.

The even number questions were unfavorable to RandomIA’s usability, while odd questions were favorable. The means and medians for questions 1, 3, 5, 7 and 9 were of higher values than the means and medians for questions 2, 4, 6, 8 and 10 (Table 1). The visualization of the results from the Likert scale from Table 1 can be found in S1 Fig.

For Q11 (“I would change my medical management based on the results provided by RandomIA after its final and validated version, such as intubating, hospitalizing, or starting intensive care early”), the mean was 2.71. The value, when compared to the median of 3 on the Likert scale, suggests that physicians would not possibly change their medical conduct based solely on the result provided. In variable Q13 (“How confident are you in the prediction information available in the application”), the proportion of positive scores (4 and 5) was higher than the proportion of negative scores (1 and 2), indicating confidence in the information contained in the application.

**Fig 3:**
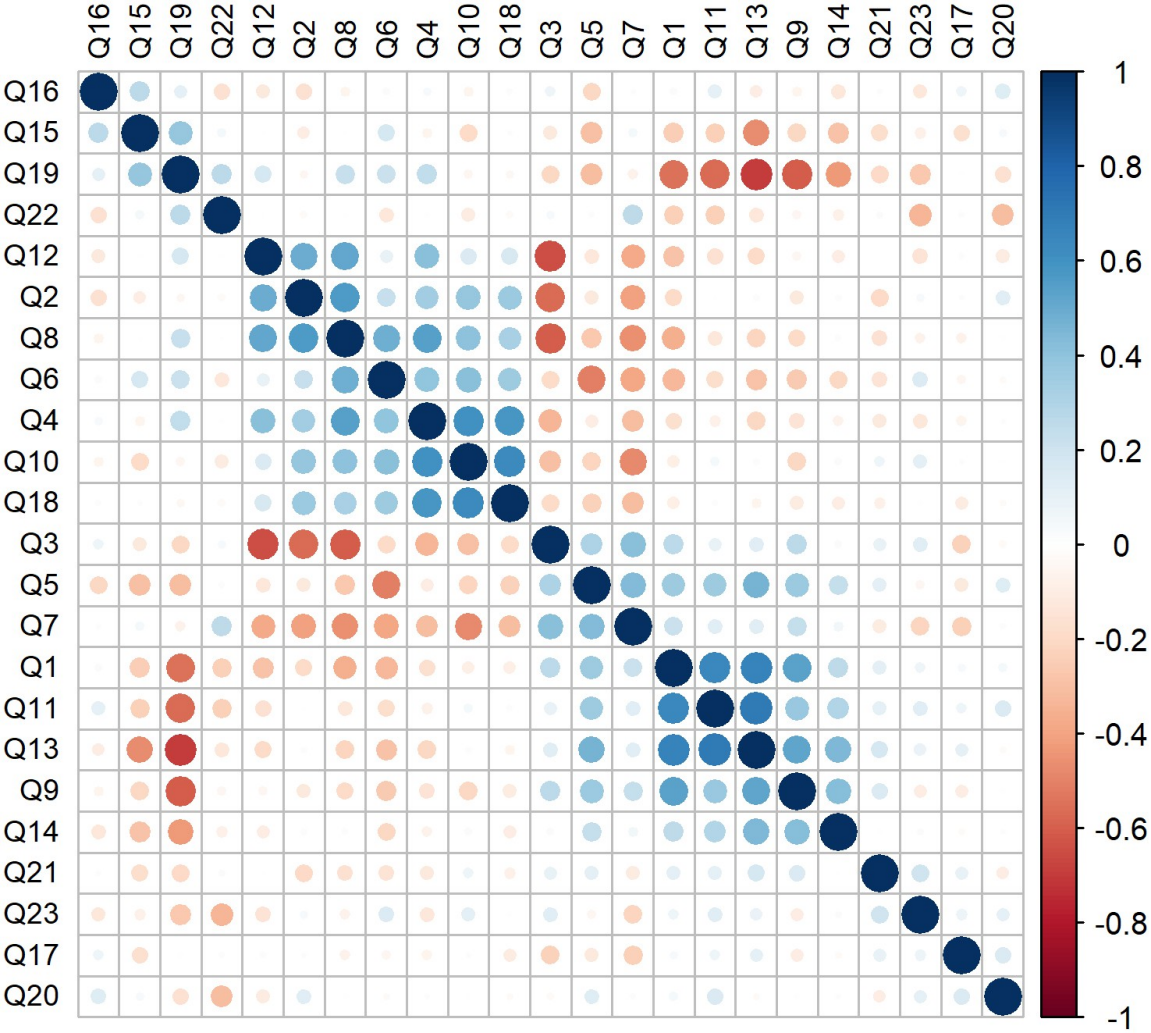
Correlation matrix between the RandomIA questionnaire questions.

Fig 3 presents the correlations between the items in the RandomIA questionnaire. In general, correlations were weak, ranging from -0.5 to 0.5. Questions Q19 with Q1, Q9, Q11, Q13 and Q14; and Q3 with Q2, Q8 and Q12 had strong correlations, but below 0.8. For detailed information on the definition of each item, see S2 Table.

**Fig 4:**
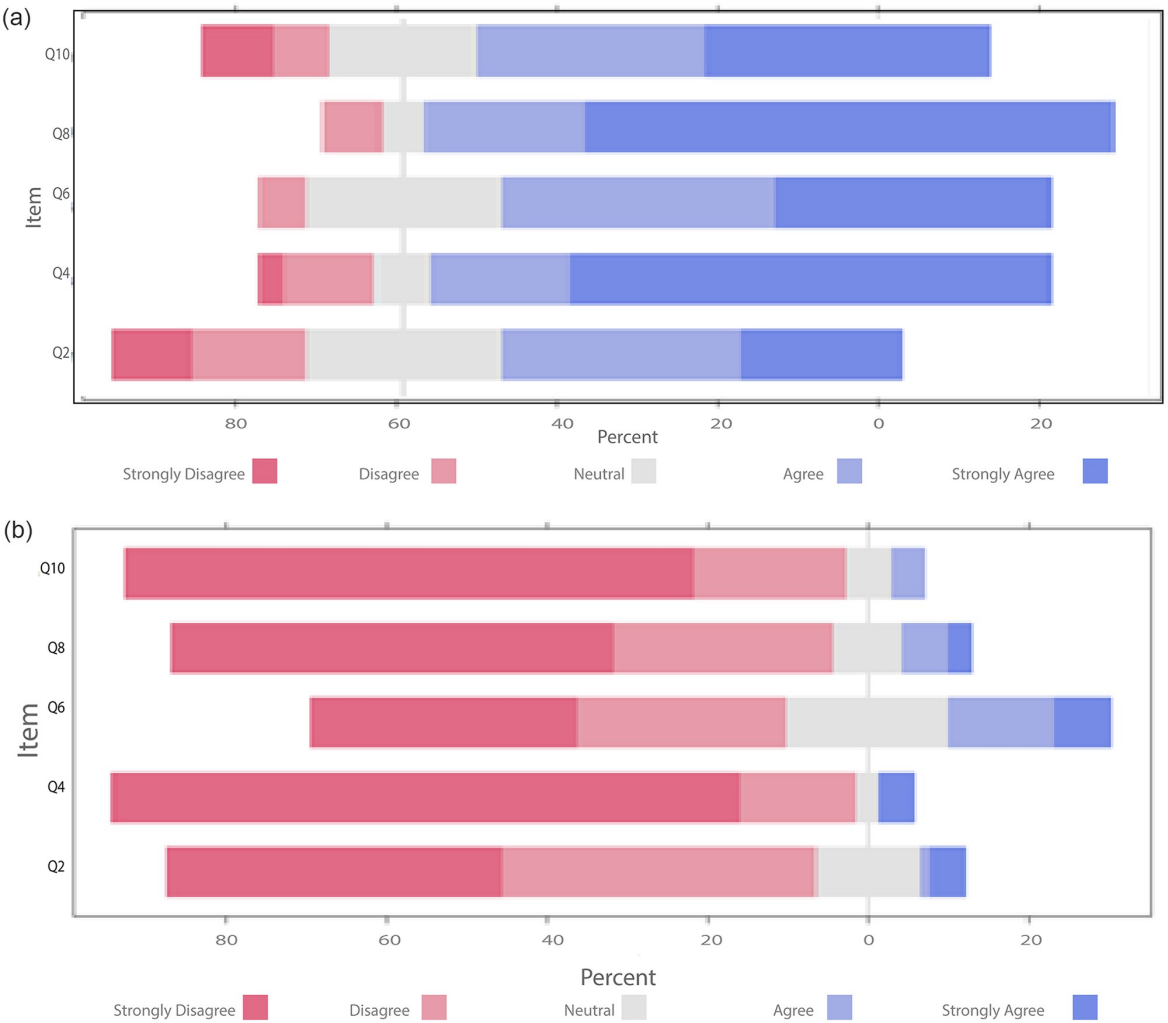
Proportions of responses to specific SUS questions in the questionnaire applied.

Fig 4a highlights the favorable responses to the usability of the application. The visualization of high grades in blue scale shows a greater proportion of these answers. On the other hand, Fig 4b shows a higher proportion of lower grades. However, this result corroborates the favorable usability of the application, since these questions, visualized in shades of red, report against the usability of the application.

Table 2 presents the proportion of responses according to each variable and their categories. In Q12 (“How do you consider using the application for customer service”), 79.7% of the participants found the application easy to use and quick to obtain the answer, while only 1.4% considered it difficult and slow to obtain. In variable Q14 ("The application’s prediction results were contrary to the diagnostic and/or prognostic impressions in their daily clinical practice"), for 42% of the participating physicians there was no divergence between the prediction and the clinical practice impressions, but 17.39% responded that there was a discrepancy with the diagnostic impression. In variable Q15 (“What do you think about the number of patient information/variables currently available in the app?”), 39.1% of professionals reported that the number of information requested was adequate, while 43.5% responded that there was a lack of relevant information.

**Table 2:** Descriptive analysis for categorical variables.

Variable	Answer	Proportion
Q12	Easy to use and quick to get the answer	79.7%
	Easy to use and slow to get the answer	8.7%
	Difficult to use and quick to get the answer	10.1%
	Difficult to use and slow to get the answer	1.4%
Q14	There was no divergence	42.0%
	Divergence with both	17.4%
	Divergence with the prognostic impression	23.2%
	Divergence with the diagnostic impression	17.4%
Q15	Missing information / important variables	43.5%
	Excessive	17.4%
	Appropriate	39.1%
Q17	Different	37.7%
	Equal	62.3%
Q17.1A	Preference 1	9.8%
	Preference 2	24.4%
	Preference 3	65.9%
Q17.1B	Preference 1	35.7%
	Preference 2	57.1%
	Preference 3	7.1%
Q17.1C	Preference 1	52.4%
	Preference 2	19.0%
	Preference 3	28.6%
Q17.3A	Preference 1	15.4%
	Preference 2	19.2%
	Preference 3	65.4%
Q17.3B	Preference 1	37.5%
	Preference 2	62.5%
Q173.C	Preference 1	42.9%
	Preference 2	32.1%
	Preference 3	25.0%
Q174.A	Preference 1	18.5%
	Preference 2	18.5%
	Preference 3	62.9%
Q174.B	Preference 1	34.6%
	Preference 2	57.6%
	Preference 3	7.6%
Q174.C	Preference 1	44.4%
	Preference 2	29.6%
	Preference 3	25.9%
Q18	Yes. I fully understood the subtitles	81.2%
	I didn’t understand the subtitles	1.4%
	Partially understood. not understood some interpretations	17.4%
Q19	Yes	69.6%
	No	30.4%

In Q17, 62.3% of participants preferred equal views of prediction results for diagnostic and prognostic outcomes, while the other 37.7% opted for different options. Among those who opted for similar visualizations, 65.9% preferred the third, i.e., the most complex. Still in this group, 13.04% indicated that their choice was motivated by: (i) the presentation was simpler and more intuitive, (ii) the presentation was visually easier to understand, (iii) the subtitles were more explanatory, and (iv) the colors helped in the interpretation of the results. Among the 37.7% who opted for visualization of different results, 65.4% preferred the more complex visualization for the diagnostic outcome results and 62.9% opted for a more complex visualization for the prognostic outcome.

In variable Q18 (“The explanation of the options was sufficient to understand the results of the predicted outcomes”), 81.2% of physicians understood the explanation provided by the description of the outcomes, while 17.4% partially understood it. Finally, for variable Q19 (“I would recommend this prediction diagnostic app for use as clinical decision support”), 69.6% of participants would recommend the prediction app for clinical decision support.

Table 3 presents the differences in proportions of the response options distributed on a Likert Scale, according to sex. There were differences between positive and negative proportions in almost all System Use Scale (SUS) questions. There was no statistically significant difference between the proportions of questions Q5 and Q8 for females. The responses were statistically favorable to the usability of the application, when using the Wilcoxon Test (Table 3).

**Table 3:** Difference in the proportions of the Likert Scale options by sex.

	F(n = 33), M(n = 36)	Low score^1^	Neutral	High score^2^	Wilcoxon P-value^3^	Multinominal P-value^4^
Q1	Female	15.15	36.36	48.48	<0.001	0.66
	Male	33.33	13.89	52.78	<0.001	
Q2	Female	87.88	9.09	3.03	<0.001	0.04
	Male	75.00	16.67	8.33	<0.001	
Q3	Female	9.09	9.09	81.82	<0.001	0.09
	Male	19.44	5.56	75.00	<0.001	
Q4	Female	93.94	6.06	0.00	0.000	0.23
	Male	91.67	0.00	8.33	<0.001	
Q5	Female	3.03	21.21	75.76	0.069	0.34
	Male	8.33	27.78	63.89	0.002	
Q6	Female	63.64	21.21	15.15	<0.001	0.91
	Male	55.56	19.44	25.00	<0.001	
Q7	Female	9.09	9.09	81.82	<0.001	0.25
	Male	5.56	2.78	91.67	0.003	
Q8	Female	90.91	6.06	3.03	0.060	0.49
	Male	75.00	11.11	13.89	<0.001	
Q9	Female	6.06	24.24	69.70	0.010	0.5
	Male	25.00	13.89	61.11	<0.001	
Q10	Female	87.88	9.09	3.03	0.030	0.53
	Male	91.67	2.78	5.56	0.001	

^1^ Combination of strongly disagree and disagree responses.

^2^ Combination of agree and strongly agree responses.

^3^ Wilcoxon test for comparison between proportions of the Low scores and High scores.

^4^ Comparison of equidistant profiles between groups using the p-value of χ2 for the likelihood ratio.

Regarding question Q5 (“I found the various functions in this system were well integrated”), despite the superiority of positive responses over the negative, there was no statistically significant difference between these proportions for females. The same occurred with Q8 (“I found the system very cumbersome to use”), in which the answers were negative when considering the female gender, but with no statistically significant difference. Overall, the profile of responses between the sexes was different. The graphical visualizations of the proportions of the Likert scale for biological sex can be found in the S2 and S3 Figs.

Table 4 presents the difference in proportions for the Likert scale options according to the age group of physicians. The age variable of physicians was separated into two groups: younger (18 to 39 years of age) and seniors (40 years old or more). The answers according to the age groups were uniform, since in the odd questions, which is about the positive usability of the application, the answers had a higher proportion of higher (positive) scores. This indicates that there was a significant difference when using the Wilcoxon test in relation to responses with a low score (negative) in both age groups (young and senior). The same behavior was observed for questions that had statements against the good usability of the application (even questions). In these, the scores were predominantly low and, therefore, contrary to the statements of low usability. In this type of questions, the Wilcoxon test detected a significant difference in all responses.

**Table 4:** Difference in the proportions of the Likert scale options by age.

	Seniors (n = 24), Younger (n = 45)	Low score^1^	Neutral	High score^2^	Wilcoxon P-value^3^	Multinominal P-value^4^
Q1	Seniors	25.00	16.67	58.33	<0.001	0.64
	Younger	24.44	28.89	46.67	<0.001	
Q2	Seniors	83.33	16.67	0.00	0.000	0.05
	Younger	80.00	11.11	8.89	<0.001	
Q3	Seniors	16.67	0.00	83.33	<0.001	0.09
	Younger	13.33	11.11	75.56	<0.001	
Q4	Seniors	91.67	4.17	4.17	0.006	0.23
	Younger	93.33	2.22	4.44	0.001	
Q5	Seniors	4.17	37.50	58.33	0.090	0.35
	Younger	6.67	17.78	75.56	0.001	
Q6	Seniors	54.17	25.00	20.83	0.006	0.91
	Younger	62.22	17.78	20.00	<0.001	
Q7	Seniors	0.00	8.33	91.67	0.000	0.25
	Younger	11.11	4.44	84.44	<0.001	
Q8	Seniors	79.17	12.50	8.33	0.010	0.48
	Younger	84.44	6.67	8.89	<0.001	
Q9	Seniors	16.67	12.50	70.83	<0.001	0.45
	Younger	15.56	22.22	62.22	<0.001	
Q10	Seniors	87.50	8.33	4.17	0.030	0.53
	Younger	91.11	4.44	4.44	0.002	

^1^ Combination of strongly disagree and disagree responses.

^2^ Combination of agree and strongly agree responses.

^3^ Wilcoxon test for comparison between proportions of the Low scores and High scores.

^4^ Comparison of equidistant profiles between groups using the p-value of χ2 for the likelihood ratio.

When the results were analyzed along the Likert scale, we found that they were parallel, i.e., that they did not present significant differences. This indicates that age did not influence the type of visualization and the response that clinicians expect from an e-health application based on a machine learning solution. The graphical visualizations of the proportions of the Likert scale for age can be found in the S4 and S5 Figs.

Table 5 presents the results according to having a medical specialty and the S7 and S8 Figs show the graphical visualizations of the proportions of their Likert scale. When comparing physicians with some type of specialty with general practitioners, we found that the overall response pattern was the same. That is, there was no statistical difference when considering the professional profiles of physicians (p-value of χ2 for the likelihood ratio). Regarding the difference in positive and negative answers for the SUS questions, we found that for the odd-numbered questions, there were differences in the proportion of high scores in relation to low scores. The proportion of high grades was higher in the odd (Q1, Q3, Q5, Q7 and Q9) and even (Q2, Q4, Q6, Q8 and Q10) items, reiterating the favorable evaluation of the application. For differences in Likert scale proportions by geographic region of Brazil, see S3A and S3B Table and S6–S11 Figs.

**Table 5:** Difference in the propositions of the Likert Scale options by medical specialty.

	G(n = 30), M.S.(n = 39)	Low score^1^	Neutral	High score^2^	Wilcoxon P-value^3^	Multinominal P-value^4^
Q1	Generalist	13.33	26.67	60.00	0.004	0.64
	Medical Specialty	33.33	23.08	43.59	0.020	
Q2	Generalist	80.00	16.67	3.33	0.060	0.05
	Medical Specialty	82.05	10.26	7.69	0.001	
Q3	Generalist	13.33	13.33	73.33	<0.001	0.09
	Medical Specialty	15.38	2.56	82.05	<0.001	
Q4	Generalist	96.67	0.00	3.33	0.030	0.22
	Medical Specialty	89.74	5.13	5.13	<0.001	
Q5	Generalist	10.00	20.00	70.00	0.002	0.35
	Medical Specialty	2.56	28.21	69.23	0.060	
Q6	Generalist	26.67	23.33	2.53	<0.001	0.92
	Medical Specialty	66.67	15.38	17.95	<0.001	
Q7	Generalist	13.33	6.67	80.00	0.004	0.23
	Medical Specialty	2.56	5.13	92.31	0.010	
Q8	Generalist	10.00	6.67	1.73	<0.001	0.47
	Medical Specialty	82.05	7.69	10.26	<0.001	
Q9	Generalist	13.33	20.00	66.67	<0.001	0.48
	Medical Specialty	17.95	17.95	64.10	<0.001	
Q10	Generalist	86.67	6.67	6.67	0.004	0.53
	Medical Specialty	92.31	5.13	2.56	0.020	

^1^ Combination of strongly disagree and disagree responses.

^2^ Combination of agree and strongly agree responses.

^3^ Wilcoxon test for comparison between proportions of the Low scores and High scores.

^4^ Comparison of equidistant profiles between groups using the p-value of χ2 for the likelihood ratio.

To identify the factors associated with the usability of the application, we performed Factor Analyses. Initially, applicability was tested using the Kaiser-Meyer-Olkin (KMO) test and a value of 0.64 was obtained (p-value >0.5). This indicates the suitability of applying the technique. Then the Bartlett sphericity test was performed to test the null hypothesis that the correlation matrix is an identity matrix, and a significance p-value < 0.01 was obtained. Then the scree-plot was performed to determine the number of values necessary to retain the greatest amount of variability (S12 Fig).

The commonalities are amounts of variances (correlations) of each variable that are explained by the factors. The greater the commonality, the greater is the explanatory power of that variable by the factor. Specificity or error is the portion of data variance that cannot be explained by the factor and is characterized by the unique proportion of the variable not shared with the others. This value is obtained by subtracting one from the commonality. The higher the specificity, the lower the relevance of a given variable in the factor model. In Table 6, the variables Q23, Q22, Q20, Q17, Q16, Q14 had less weight in the factor analysis.

**Table 6:** List of factor specificity values.

**Q1**	**Q2**	**Q3**	**Q4**	**Q5**	**Q6**	**Q7**	**Q8**
0,326	0,343	0,214	0,101	0,099	0,514	0,358	0,373
**Q9**	**Q10**	**Q11**	**Q12**	**Q13**	**Q14**	**Q15**	**Q16**
0,005	0,29	0,288	0,327	0,11	0,72	0,577	0,711
**Q17**	**Q18**	**Q19**	**Q20**	**Q21**	**Q22**	**Q23**	
0,797	0,444	0,244	0,691	0,724	0,509	0,609	

Source: RandomIA app survey data. Prepared by the authors.

**Fig 5:**
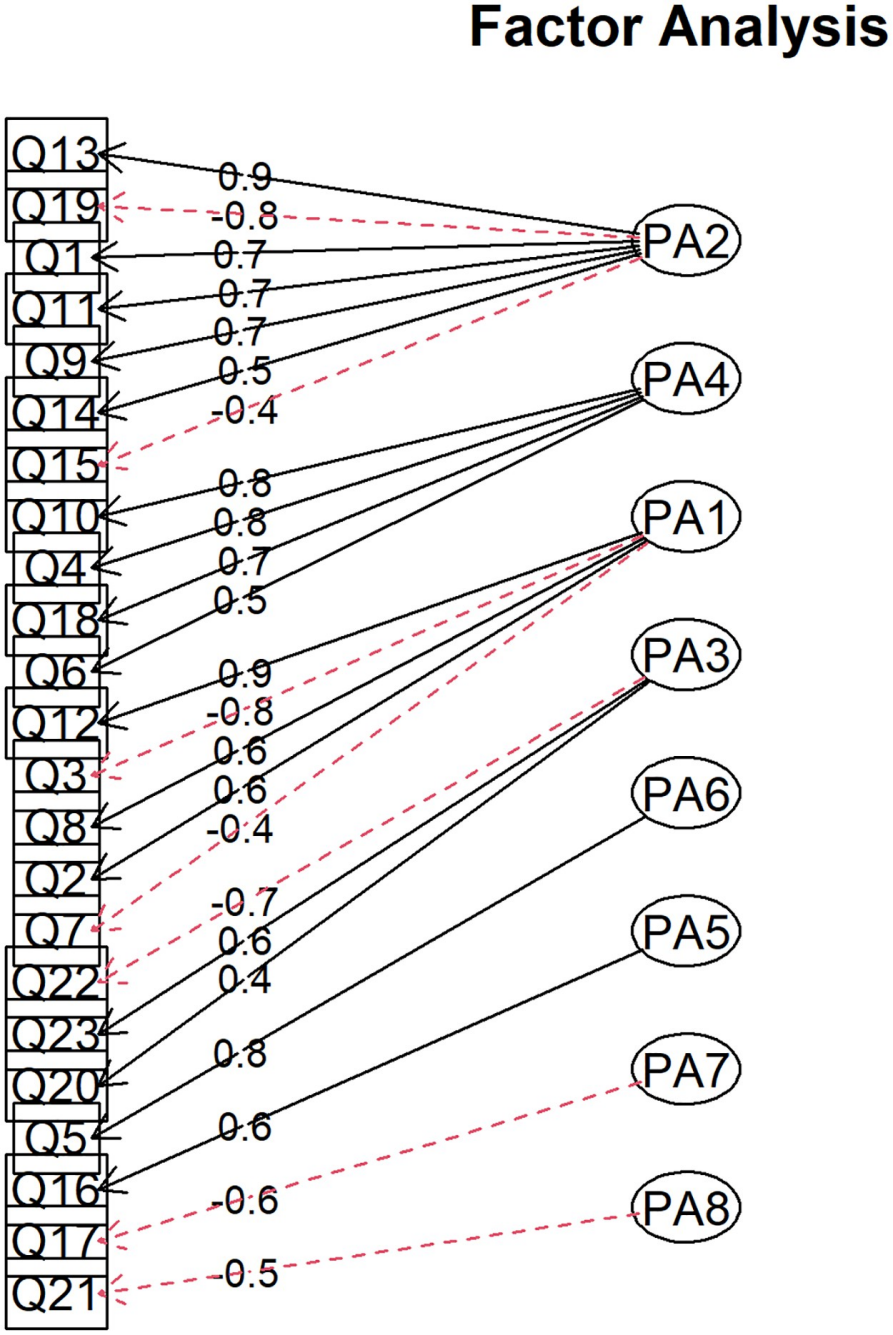
Factor analysis with loadings and factors associated with each item in the RandomIA questionnaire.

The variables that make up each factor are shown in Fig 5. Factor 3 represented by PA1 is composed of positive loadings in Q12, Q8, Q2 and negative in Q3 and Q7. This factor involves variables regarding the use of the application, i.e., the usability factor. Factor 4 (PA2) is composed of the items (Q13, Q1, Q11, Q9 and Q14) in a positive way and Q19 and Q15 in a negative way. This factor can be considered a measure of confidence in the use of the application. Factor 5 (PA3) is composed of variables Q20 and Q23 with positive loadings, and Q22 with negative loading. This combination is related to the characteristics of the physicians participating in the study. Factor 4 has questions Q10, Q4, Q18 and Q6, a factor related to difficulties in using the application. Factor 5 is linked to the amount of application outcomes. Factor 6 refers to the integration of application functions. Factor 7, of great relevance to the outcome of the present study, refers to the type of preference for viewing outcomes. Finally, Factor 8 is related to age.

The principal component analyzes and biplots to verify the association between the questions and the physicians, based on the RandomIA questionnaire, can be found in S4 and S5 Tables, S13 and S14 Figs.

Biplot analysis by subgroups helped in the evaluation of the robustness of the results found for the RandomIA questionnaire. Such representations usually allow the visualization of vectors represented by the questions, in which the size of these vectors is associated with the importance of these items, while ellipses are representations of the area covered by these questions within each group.

**Fig 6:**
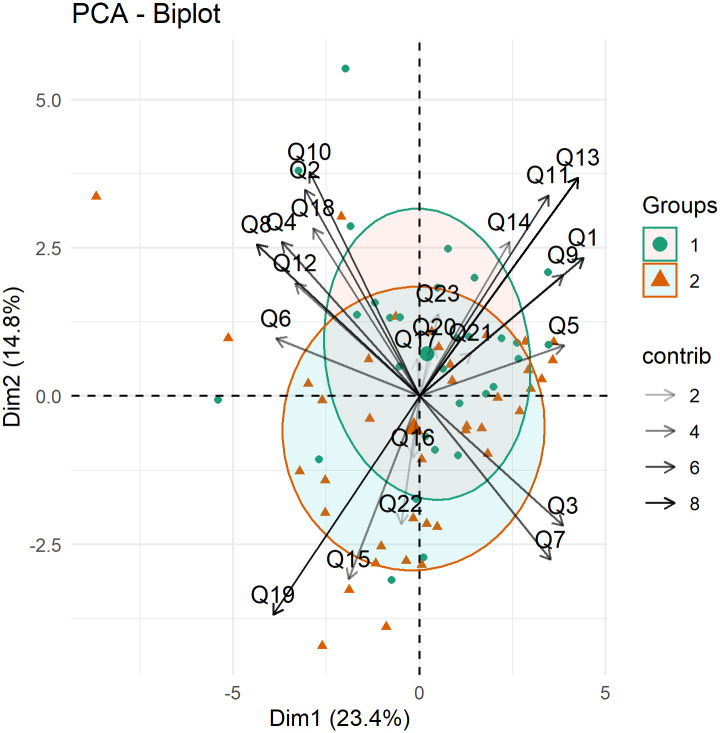
Biplot by medical specialty to visualize the association between items in the first two dimensions from the RandomIA questionnaire by presence of a medical specialty.

An analysis by the presence or absence of a medical specialty is represented by the number 1 for general practitioners and 2 for specialist doctors (Fig 6). There was no difference between the groups since the ellipses are superimposed. Thus, it was not possible to analyze which specialty has greater acceptance of the application.

**Fig 7:**
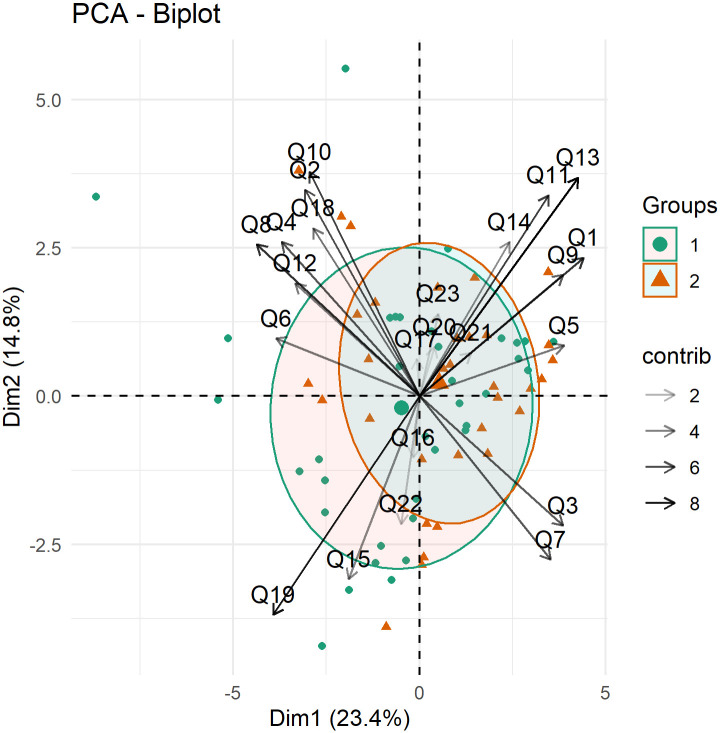
Biplot by gender of the responding physicians to visualize the association between items in the first two dimensions from the RandomIA questionnaire.

Regarding the sex of the participants, the association represented by the biplot in Fig 7 indicates that there was no difference between the groups, which is both men (group 1) and women (group 2) are superimposed for the questions.

The differences between the regions of the country are represented by S15 Fig. Through the techniques shown, it was possible to reduce a 23-question questionnaire to eight latent variables with factor analysis. Such a tool is useful for summarizing information obtained from questionnaires applied to the most diverse subjects. It was also possible to identify the factors giving them a practical meaning. These results corroborated the findings of the Likert scale.

## Discussion

The study found a high physician adherence to a ML-based application for predicting health outcomes. More importantly, the questions designed to test the usability of the application proved useful for this purpose. We also found that most physicians preferred a more complex form of visualization for receiving the final predictions generated by the machine learning algorithm.

There have been some previous proposals for mobile applications based on ML, all developed from 2016 onwards [5, 22–26]. The limitations listed by these studies include insufficient observations from physicians, lack of publicly available data, unavailability of internet connection in remote areas and lack of reliability of sources that provided the data sets. However, the interaction between users, in this case the medical community, and the results provided by the algorithms are rarely discussed as a potential limitation of the use of such ML models. The difficulties of implementing a machine learning model in clinical practice are not only due to technical difficulties in developing the algorithms, but may be also due to ethical difficulties [27] and the diversity of users and developers involved [28], among other challenges.

This study had some limitations. As this is a new area of research, previous questionnaires capable of evaluating the usability of ML-based health applications were not identified in the literature, so we had to adapt SUS for this specific purpose. Another limitation is that a convenience sampling was the only technique available to collect data among participants of the network. Lastly, the high proportion of negative results in Q11 (“I would change my medical conduct based on the results provided by RandomIA after its final and validated version, e.g., intubation or not hospitalized or not starting intensive care early, etc.”), may be since due to the fact that the actual algorithm used to provide the prediction has not yet been properly calibrated for accurate assertiveness regarding COVID-19 prognostic results.

## Conclusions

Our study was the first to assess how healthcare professionals prefer to receive the results of ML-based applications. The main results from the study can be extended to the development of other e-health technologies and the improvement of already existing ones, as it may help in the understanding of discouraging factors for users and in the better knowledge of the target audience of new AI applications.

## Supporting information

S1 Table Variables used in predictive models. (DOCX)

S2 Table Questionnaire for RandomIA. (DOCX)

S3 Table **A**. Difference in the proportions of the Likert Scale options by Brazil region for the first five questions. **B**. Difference in the proportions of the Likert Scale by Brazil region for the last five questions.
(ZIP)

S4 Table Details of factors obtained through Principal Component Analysis. (DOCX)

S5 Table Principal Component Analysis results. (DOCX)

S1 Fig Choropleth representation of proportions on the Likert scale. (DOCX)

S2 Fig Barplot proportions of the Likert Scale options by biological sex for odds questions. (DOCX)

S3 Fig Barplot proportions of the Likert Scale options by biological sex for even questions. (DOCX)

S4 Fig Barplot proportions of the Likert Scale options by age group for odds questions. (DOCX)

S5 Fig Barplot proportions of the Likert Scale options by age group for even questions. (DOCX)

S6 Fig Barplot proportions of the Likert Scale options by medical speciality for odds questions. (DOCX)

S7 Fig Barplot proportions of the Likert Scale options by medical speciality for even questions. (DOCX)

S8 Fig Barplot proportions of the Likert Scale options by Brazil regions for odds questions (Q1, Q3, Q5). (DOCX)

S9 Fig Barplot proportions of the Likert Scale options by Brazil regions for odds questions (Q7, Q9). (DOCX)

S10 Fig Barplot proportions of the Likert Scale options by Brazil regions for even questions (Q2, Q4, Q6). (DOCX)

S11 Fig Barplot proportions of the Likert Scale options by Brazil regions for even questions (Q8, Q10). (DOCX)

S12 Fig Scree-plot of the eigenvalues sorted in descending order for the RandomIA questionnaire data. (DOCX)

S13 Fig Biplot showing the association between items plotted in the first two dimensions from the RandomIA questionnaire. (DOCX)

S14 Fig Biplot showing the dispersion of doctors in relation to the questions in the first two dimensions, based on the RandomIA questionnaire. (DOCX)

S15 Fig Biplot showing association of the questions in the first two dimensions from the RandomIA questionnaire by Brazilian regions. (DOCX)

S1 Dataset (XLSX)
