# Supplementary material for: Physician preference for receiving machine learning predictive results: A cross-sectional multicentric study
Source: PLoS One. 2022 Dec 14;17(12):e0278397. doi: 10.1371/journal.pone.0278397 (PMC9749966; doi:10.1371/journal.pone.0278397)
Supplement: S1 Table — (DOCX) [file pone.0278397.s001.docx]

**S1 Table. Variables used in predictive models.**

| # | Variable | Description |  |  |  |
| --- | --- | --- | --- | --- | --- |
| 1 | **Origin_pcr_covid** | CRP exam result source |  |  |  |
| 2 | **ID_PATIENT** | Unique patient identification (anonymized/de-identified variable) |  |  |  |
| 3 | **RT_PCR_COVID** | RT-PCR result for covid-19 |  |  |  |
| 4 | **DATE_RT_PCR** | RT-PCR exam order date |  |  |  |
| 5 | **TIME_DAYS** | Length of stay of the patient in the hospital (days) |  |  |  |
| 6 | **DATE_ADM** | Date of admission to a common bed (if applicable) |  |  |  |
| 7 | **DATE_ICU_ADM** | Date of ICU admission (if applicable) |  |  |  |
| 8 | **DATE_OT** | Start date of mechanical ventilation (if applicable) |  |  |  |
| 9 | **DATE_DEATH** | Date of death (if applicable) |  |  |  |
| 10 | **DATE_DISC** | Date of discharge from hospital (if applicable) |  |  |  |
| 11 | **DATE_DIET_PAR** | Date the patient received parenteral diet (if applicable) |  |  |  |
| 12 | **DATE_DRUGS** | Date the patient received vasoactive drug (if applicable) |  |  |  |
| 13 | **DATE_HD** | Date the patient received dialysis (if applicable) |  |  |  |
| 14 | **DATE_PALIATE** | Date the patient received palliative care (if applicable) |  |  |  |
| 15 | **AGE** | Patient age (years) |  |  |  |
| 16 | **GENDER** | Patient's gender |  |  |  |
| 17 | **RACE** | Race |  |  |  |
| 18 | **BRADEN** | Risk scale of developing pressure ulcers (number) |  |  |  |
| 19 | **HR** | Heart rate (bpm) |  |  |  |
| 20 | **RR** | Respiratory rate (bpm) |  |  |  |
| 21 | **BP_SIST** | Systolic Blood Pressure (mmHg) |  |  |  |
| 22 | **BP_DIAST** | Diastolic Blood Pressure (mmHg) |  |  |  |
| 23 | **MAP** | Mean arterial pressure MAP = DBP + [0.333 x (bp_sist - bp_diast)] (mmHg) |  |  |  |
| 24 | **TEMP** | Body temperature (degrees Celsius) |  |  |  |
| 25 | **SAT** | Oxygen Saturation SaO2 (percentage) |  |  |  |
| 26 | **WEIGHT** | Patient weight (kilograms) |  |  |  |
| 27 | **HEIGHT** | Height in centimeters (cm) |  |  |  |
| 28 | **BMI** | Body mass index (Kg/m2) |  |  |  |
| 29 | **HB** | Hemoglobin (g/dL) |  |  |  |
| 30 | **HT** | Hematocrit (percentage of red blood cells) |  |  |  |
| 31 | **PLT** | Platelets (quantity/mm3) |  |  |  |
| 32 | **RBC_ABS** | Red blood cells (quantity/mm3) |  |  |  |
| 33 | **MCHC** | Mean Corpuscular Hemoglobin Concentration |  |  |  |
| 34 | **MCH** | Mean Corpuscular Hemoglobin (pg) |  |  |  |
| 35 | **RDW** | Red Cell Distribution Width (%) |  |  |  |
| 36 | **MCV** | Mean corpuscular volume (fL) |  |  |  |
| 37 | **WBC_ABS** | Leukocytes (quantity/mm3) |  |  |  |
| 38 | **SEG_ABS** | Neutrophils (quantity/mm3) |  |  |  |
| 39 | **LYMPH_ABS** | Lymphocytes (quantity/mm3) |  |  |  |
| 40 | **NEUT_LYMPH** | Ratio of neutrophils to lymphocytes (neutrophils/lymphocytes) |  |  |  |
| 41 | **LYMPH_CRP** | Lymphocyte to C-Reactive Protein Ratio (lymphocytes/C-Reactive Protein) |  |  |  |
| 42 | **BASO_ABS** | Basophils (quantity/mm3) |  |  |  |
| 43 | **EOS_ABS** | Eosinophils (amount/mm3) |  |  |  |
| 44 | **MONO_ABS** | Monocytes (quantity/mm3) |  |  |  |
| 45 | **CRP** | C Reactive Protein (mg /dL) |  |  |  |
| 46 | **ALBUMIN** | 3,5 - 5 g/dL (g/dL) |  |  |  |
| 47 | **DHL** | Lactate dehydrogenase (U/L) |  |  |  |
| 48 | **TGP** | Glutamic-pyruvic transaminase enzyme 7 - 56 U/L (U/L) |  |  |  |
| 49 | **TGO** | Enzyme glutamic-oxalacetic transaminase 5-40 U/L (units per liter of serum) |  |  |  |
| 50 | **BILI_TOTAL** | Total bilirubin mg/dL (milligrams per deciliter) |  |  |  |
| 51 | **BILI_DIRECT** | Direct bilirubin mg/dL (milligrams per deciliter) |  |  |  |
| 52 | **BILI_INDIRECT** | Indirect bilirubin mg/dL (milligrams per deciliter) |  |  |  |
| 53 | **UREIA** | Urea (mg /dL) |  |  |  |
| 54 | **SODIO** | Sodium (mEq/L) |  |  |  |
| 55 | **POTASSIO** | Potassium (mEq/L) |  |  |  |
| 56 | **CREATININA** | Creatinine (mg/dL) |  |  |  |
| 57 | **TROPONINA** | Troponin (mcg/L (micrograms per Liter) |  |  |  |
| 58 | **D_DIMEROS** | ng/dL |  |  |  |
| 59 | **BNP** | Brain natriuretic peptide 0 -70 pg/ml (picogram/milliliter) |  |  |  |
| 60 | **LACTATO_VENOSO** | mmol/L (milimol por Litro) |  |  |  |
| 61 | **FIBRINOGENIO** | mg/dL (miligramas por decilitro) |  |  |  |
| 62 | **CPK** | Creatine Phosphokinase U/L (Units per Liter) |  |  |  |
| 63 | **CKMB** | Creatine kinase U/L (Units per Liter) |  |  |  |
| 64 | **INR** | International Normalized Ratio Relationship between a patient's prothrombin time and a standard prothrombin time value |  |  |  |
| 65 | **TTPA** | Activated partial thromboplastin time (seconds) |  |  |  |
| 66 | **LACTATO_ARTERIAL** | Arterial lactate mmol/L (millimol per Liter) |  |  |  |
| 67 | **GASO_PH** | Arterial blood gas analysis PH (unit) |  |  |  |
| 68 | **GASO_PO2** | Gasometry pO2 (mmHg) |  |  |  |
| 69 | **GASO_PCO2** | Gasometry pCO2 (mmHg) |  |  |  |
| 70 | **GASO_HCO3** | Plasma bicarbonate (mmol/L) |  |  |  |
| 71 | **GASO_EB** | Arterial blood gas Excess base (unit) |  |  |  |
| 72 | **GASO_SO2** | SO2 arterial blood gas analysis (percentage) |  |  |  |
| 73 | **MAGNESIO** | Serum magnesium level 1.7-2.6 mg/dL (0.7-1.1 mmol/L) |  |  |  |
| 74 | **CALCIO_IONICO** | 4,7-5,2 mg/dL (1,17-1,30 mmol/L) |  |  |  |
| 75 | **CALCIO_TOTAL** | 8.5-10.5 mg/dl (milligrams per deciliter) |  |  |  |
| 76 | **GLICOSE** | Blood glucose level 70 - 99 mg/dL (milligrams per deciliter) |  |  |  |
