# Supplementary material for: Physician preference for receiving machine learning predictive results: A cross-sectional multicentric study
Source: PLoS One. 2022 Dec 14;17(12):e0278397. doi: 10.1371/journal.pone.0278397 (PMC9749966; doi:10.1371/journal.pone.0278397)
Supplement: S2 Table — (DOCX) [file pone.0278397.s002.docx]

**S2 Table. Questionnaire for RandomIA.**

| n | | Question | Possible answers | |
| --- | --- | --- | --- | --- |
| 1 | | I think that I would like to use this system frequently | 1-5, Strongly disagree to Strongly agree | |
| 2 | | I found the system unnecessarily complex | 1-5, Strongly disagree to Strongly agree | |
| 3 | | I thought the system was easy to use. | 1-5, Strongly disagree to Strongly agree | |
| 4 | | I think that I would need the support of a technical person to be able to use this system. | 1-5, Strongly disagree to Strongly agree | |
| 5 | | I found the various functions in this system were well integrated. | 1-5, Strongly disagree to Strongly agree | |
| 6 | | I thought there was too much inconsistency in this system. | 1-5, Strongly disagree to Strongly agree | |
| 7 | | I would imagine that most people would learn to use this system very quickly. | 1-5, Strongly disagree to Strongly agree | |
| 8 | | I found the system very cumbersome to use. | 1-5, Strongly disagree to Strongly agree | |
| 9 | | I felt very confident using the system. | 1-5, Strongly disagree to Strongly agree | |
| 10 | | I needed to learn a lot of things before I could get going with this system. | 1-5, Strongly disagree to Strongly agree | |
| 11 | | I would change my medical behavior based on the results provided by the app. Ex: intubate or not, hospitalize or not, transfer to intensive care unit early etc. | 1-5, Strongly disagree to Strongly agree | |
| 12 | | How do you consider the use of the app for use during the medical shift or clinical evaluation? | Easy to use and to get the answer / Easy to use and slow to get the answer / Hard to use and quick to get the answer / Difficult to use and slow to get the answer | |
| 13 | | How much do you trust the predictions available in the app? | 1-5, Unreliable to Reliable | |
| 14 | | Were the app's prediction results contrary to the diagnostic and/or prognostic impressions in your daily clinical practice? | Divergence with diagnostic impression / Divergence with the prognostic impression / Disagreement with both / There was no divergence | |
| 15 | | What do you think about the number of patient information/variables currently available in the app? | Excessive / Adequate / Some important information/variables are missing | |
|  | | If your answer to question 15 was "Important information/variables are missing", what would they be? |  | |
| 16 | Do you consider the number of prognostic outcomes (death, ICU admission and need for mechanical ventilation) currently available in the application to be sufficient? | | | Yes / No |
|  | If you answer "No" to question 16, please add the outcomes: | | |  |
| 17 | Do you prefer the same or different views/options for diagnostic and prognostic outcomes? | | | Same / Different |
| 17.1 | If you answered "Same" in question 17, what is your order of preference? | | |  |
| 17.2 | If your answer was "Same" in question 17, please select all the options that justify the answer above (17.1) | | | The form of presentation is simpler and more intuitive / The form of presentation is visually easier to understand / Subtitles are more explanatory / The colors help me interpret the results / I prefer more elaborate or detailed views |
| 17.3 | If you answered "Different" to question 17, what is your order of preference for the DIAGNOSTIC outcome? | | |  |
| 17.4 | If you answered "Different" to question 17, what is your order of preference for PROGNOSTIC outcomes? | | |  |
| 18 | Was the explanation about each option sufficient to understand the outcome/prediction results? | | | Yes, I fully understood the subtitle/interpretation / Partially understood, I didn't understand some interpretations / I didn't understand the subtitles/interpretations |
| 19 | Would you recommend this diagnostic/prognostic prediction app for use in clinical decision support? | | | Yes / No |
|  | If you answered “No” to question 19, please state why. | | |  |
| 20 | Would you have any comments on the RandomIA app or suggestions for improvements? | | |  |
