# Supplementary material for: Physician preference for receiving machine learning predictive results: A cross-sectional multicentric study
Source: PLoS One. 2022 Dec 14;17(12):e0278397. doi: 10.1371/journal.pone.0278397 (PMC9749966; doi:10.1371/journal.pone.0278397)
Supplement: S3 Table — A. Difference in the proportions of the Likert Scale options by Brazil region for the first five questions. B. Difference in the proportions of the Likert Scale by Brazil region for the last five questions. (ZIP) [file pone.0278397.s003.zip › S3B_Table.docx]

**S3B Table. Difference in the proportions of the Likert Scale by Brazil region for the last five questions.**

|  | N(9),NE(5),CO(12),SE(34) S(9) | Low score^1^ | Neutral | High score^2^ | Wilcoxon P-value^3^ |
| --- | --- | --- | --- | --- | --- |
| Q6 | North | 44.44 | 22.22 | 33.33 | 0.03 |
|  | Northest | 80.00 | 20.00 | 0.00 | 0 |
|  | Central West | 50.00 | 25.00 | 25.00 | 0.02 |
|  | Southest | 58.82 | 20.59 | 20.59 | <0.001 |
|  | South | 77.78 | 11.11 | 11.11 | 0.08 |
| Q7 | North | 22.22 | 11.11 | 66.67 | 0.03 |
|  | Northest | 0.00 | 0.00 | 100.00 | 0 |
|  | Central West | 16.67 | 8.33 | 75.00 | 0.02 |
|  | Southest | 2.94 | 5.88 | 91.18 | 0.03 |
|  | South | 0.00 | 0.00 | 100.00 | 0 |
| Q8 | North | 77.78 | 22.22 | 0.00 | 0 |
|  | Northest | 100.00 | 0.00 | 0.00 | 0 |
|  | Central West | 83.33 | 8.33 | 8.33 | 0.11 |
|  | Southest | 85.29 | 2.94 | 11.76 | <0.001 |
|  | South | 66.67 | 22.22 | 11.11 | 0.11 |
| Q9 | North | 11.11 | 33.33 | 55.56 | 0.20 |
|  | Northest | 20.00 | 0.00 | 80.00 | 0.26 |
|  | Central West | 25.00 | 25.00 | 50.00 | 0.02 |
|  | Southest | 14.71 | 17.65 | 67.65 | <0.001 |
|  | South | 11.11 | 11.11 | 77.78 | 0.13 |
| Q10 | North | 77.78 | 11.11 | 11.11 | 0.13 |
|  | Northest | 100.00 | 0.00 | 0.00 | 0 |
|  | Central West | 91.67 | 8.33 | 0.00 | 0 |
|  | Southest | 88.24 | 5.88 | 5.88 | <0.001 |
|  | South | 100.00 | 0.00 | 0.00 | 0 |

Note: ^1^ Combination of strongly disagree and disagree responses; ^2^ Combination of agree and strongly agree responses; ^3^ Wilcoxon test for comparison between proportions of the Low scores and High scores.
