# Supplementary material for: Physician preference for receiving machine learning predictive results: A cross-sectional multicentric study
Source: PLoS One. 2022 Dec 14;17(12):e0278397. doi: 10.1371/journal.pone.0278397 (PMC9749966; doi:10.1371/journal.pone.0278397)
Supplement: S4 Table — (DOCX) [file pone.0278397.s004.docx]

**S4 Table. Details of factors obtained through Principal Component Analysis.**

| **Charges** | **Factor 1/PA2** | **Factor 2/ PA4** | **Factor 3/PA1** | **Factor 4/PA6** | **Factor 5/PA3** | **Factor 6/PA5** | **Factor 7/PA8** | **Factor 8/PA7** |
| --- | --- | --- | --- | --- | --- | --- | --- | --- |
| **SS loading** | 3,556 | 2,625 | 2,579 | 1,164 | 1,163 | 0,879 | 0,864 | 0,796 |
| **Proportion var** | 0,155 | 0,114 | 0,112 | 0,051 | 0,051 | 0,038 | 0,038 | 0,035 |
| **Cumulative var** | 0,155 | 0,269 | 0,381 | 0,431 | 0,482 | 0,52 | 0,558 | 0,592 |

Note^1^: In this case, eight factors were sufficient to explain 60% of the data variability.
