# Supplementary material for: Physician preference for receiving machine learning predictive results: A cross-sectional multicentric study
Source: PLoS One. 2022 Dec 14;17(12):e0278397. doi: 10.1371/journal.pone.0278397 (PMC9749966; doi:10.1371/journal.pone.0278397)
Supplement: S5 Table — (DOCX) [file pone.0278397.s005.docx]

**S5 Table. Principal Component Analysis Results.**

| **No. of eigenvalues#** | **Eigenvalues** | **Proportion of variance** | **Cumulative of variance**. |
| --- | --- | --- | --- |
| 1 | 5.37 | 23.35 | 23.35 |
| 2 | 3.41 | 14.82 | 38.18 |
| 3 | 1.9 | 8.25 | 46.42 |
| 4 | 1.55 | 6.72 | 53.15 |
| 5 | 1.45 | 6.31 | 59.46 |
| 6 | 1.21 | 5.24 | 64.7 |
| 7 | 01.08 | 4.68 | 69.38 |
| 8 | 0.97 | 4.22 | 73.6 |
| 9 | 0.8 | 3.49 | 77.09 |
| 10 | 0.73 | 3.19 | 80.28 |
| 11 | 0.66 | 2.86 | 83.14 |
| 12 | 0.62 | 2.7 | 85.84 |
| 13 | 0.56 | 2.45 | 88.29 |
| 14 | 0.51 | 2.2 | 90.49 |
| 15 | 0.38 | 1.63 | 92.12 |
| 16 | 0.36 | 1.57 | 93.69 |
| 17 | 0.31 | 1.33 | 95.01 |
| 18 | 0.27 | 1.18 | 96.19 |
| 19 | 0.26 | 1.11 | 97.31 |
| 20 | 0.19 | 0.83 | 98.14 |
| 21 | 0.16 | 0.69 | 98.83 |
| 22 | 0.15 | 0.64 | 99.47 |
| 23 | 0.12 | 0.53 | 100 |

Note: Principal component analysis was also used to verify which items were most associated with each research group (physicians, gender, and region). Of the eigenvalues, the first seven had variability equivalent to information on the variance of an original variable. The accumulated variance corresponds to 73.60%. It is important to emphasize that the difference in explained variability between the factorial and principal component analyzes is related to what type of variability each analysis explains.
