# Supplementary figures and images for: Physician preference for receiving machine learning predictive results: A cross-sectional multicentric study
Source: PLoS One. 2022 Dec 14;17(12):e0278397. doi: 10.1371/journal.pone.0278397 (PMC9749966; doi:10.1371/journal.pone.0278397)

**S1 Fig. Choropleth representation of proportions on the Likert scale.**


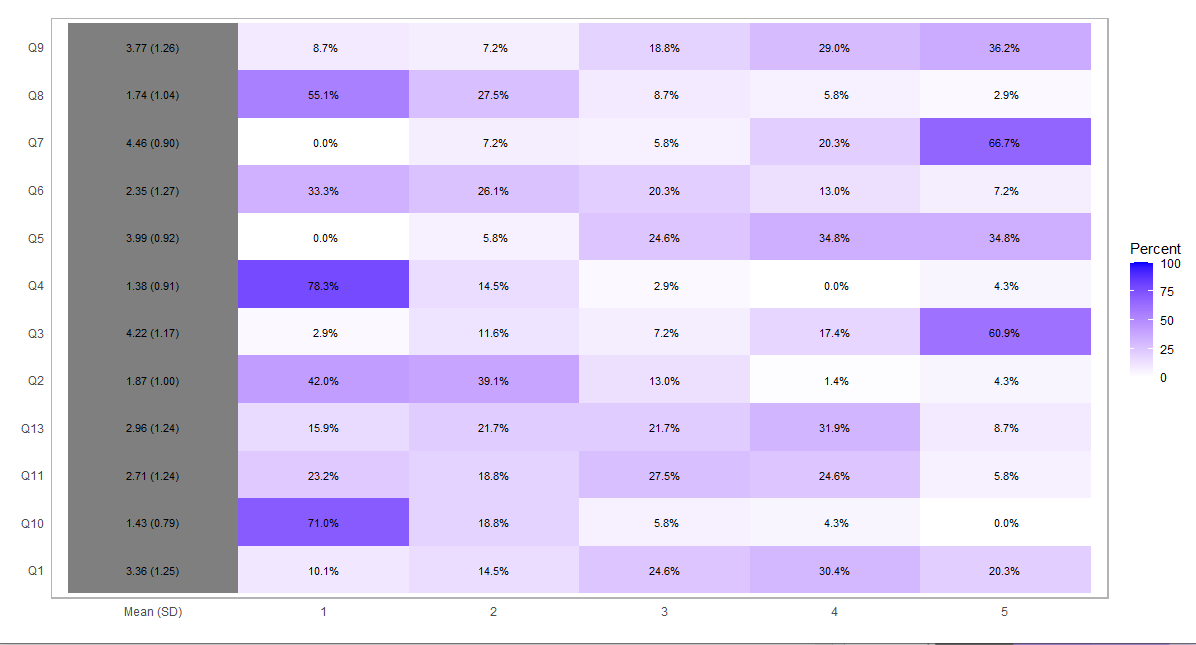

Supplement: S1 Fig — (DOCX) [file pone.0278397.s006.docx]

**S2 Fig. Barplot proportions of the Likert Scale options by biological sex for odds questions.**


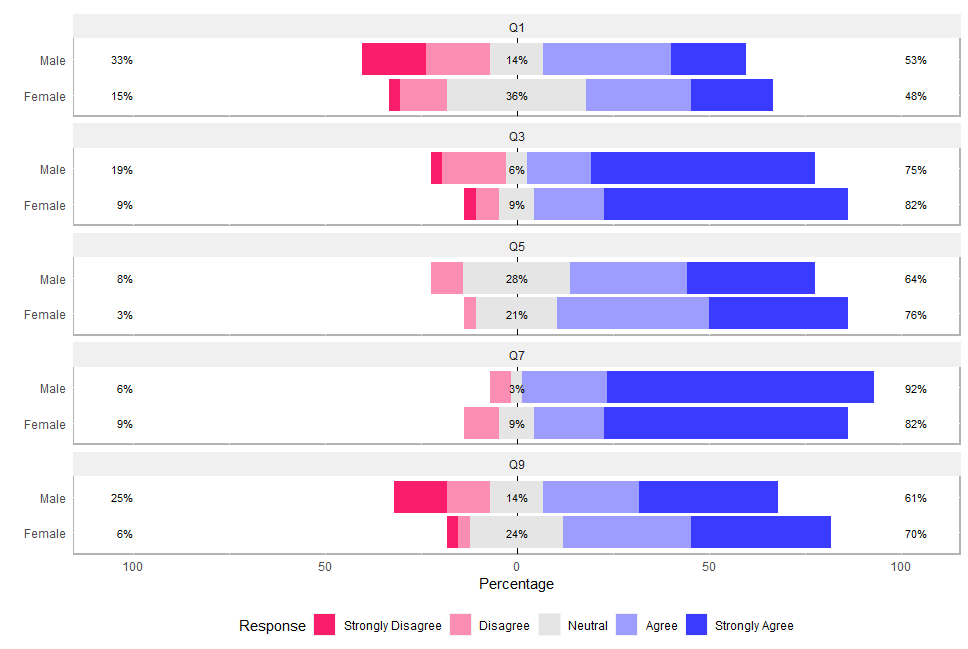

Supplement: S2 Fig — (DOCX) [file pone.0278397.s007.docx]

**S3 Fig. Barplot proportions of the Likert Scale options by biological sex for even questions.**


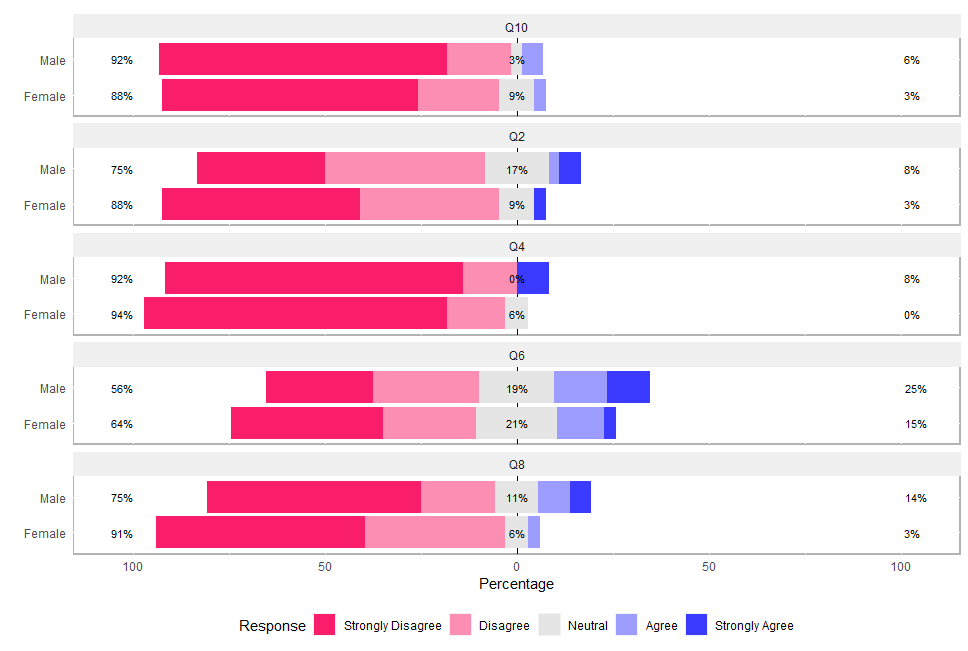

Supplement: S3 Fig — (DOCX) [file pone.0278397.s008.docx]

**S4 Fig. Barplot proportions of the Likert Scale options by age group for odds questions.**


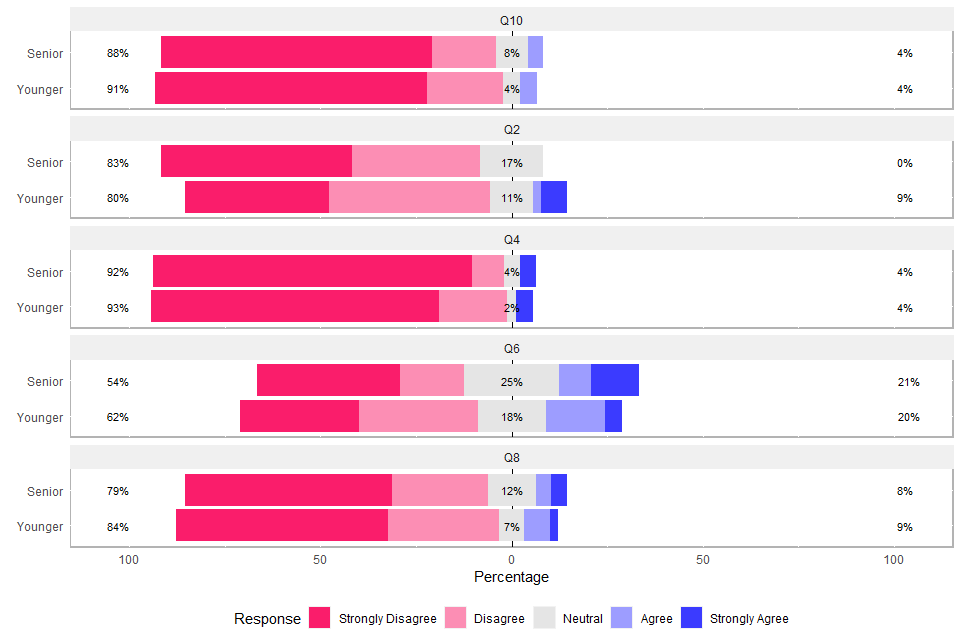

Supplement: S4 Fig — (DOCX) [file pone.0278397.s009.docx]

**S5 Fig. Barplot proportions of the Likert Scale options by age group for even questions.**


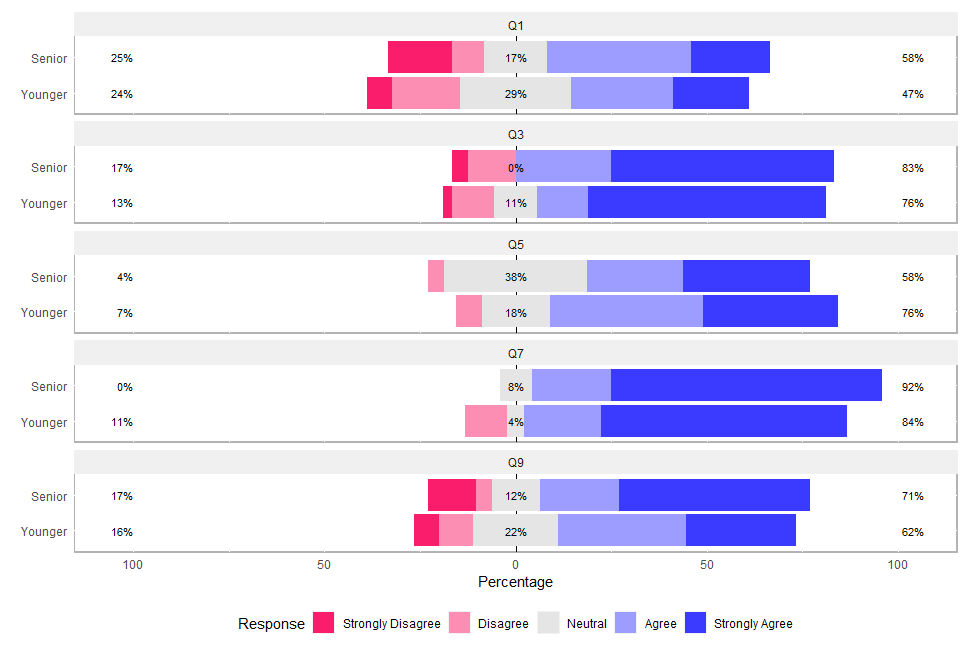

Supplement: S5 Fig — (DOCX) [file pone.0278397.s010.docx]

**S8 Fig. Barplot proportions of the Likert Scale options by Brazil regions for odds questions (Q1, Q3, Q5).**


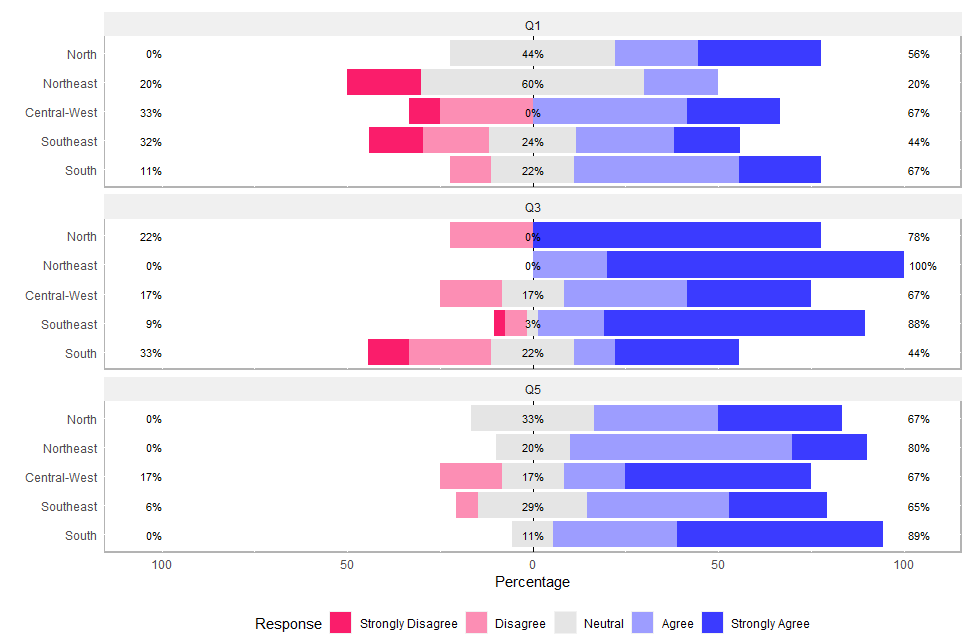

Supplement: S8 Fig — (DOCX) [file pone.0278397.s013.docx]

**S9 Fig. Barplot proportions of the Likert Scale options by Brazil regions for odds questions (Q7, Q9).**


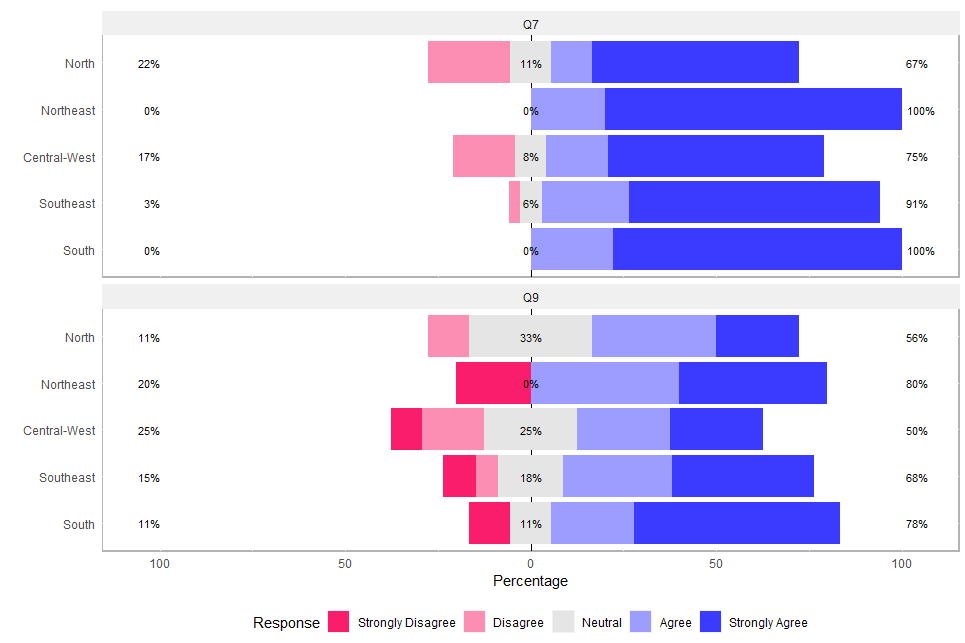

Supplement: S9 Fig — (DOCX) [file pone.0278397.s014.docx]

**S10 Fig. Barplot proportions of the Likert Scale options by Brazil regions for even questions (Q2, Q4, Q6).**


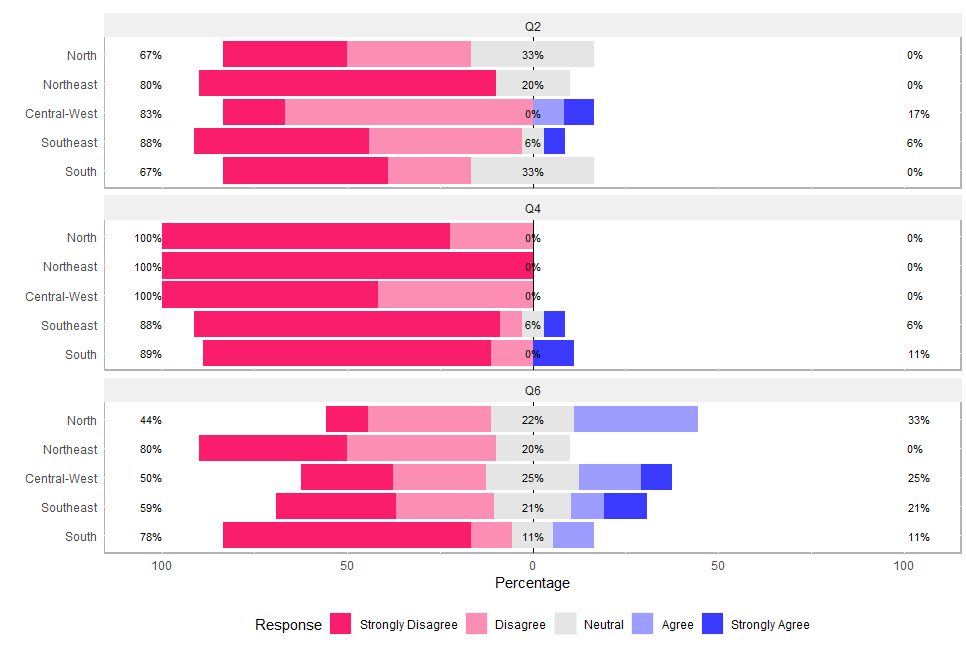

Supplement: S10 Fig — (DOCX) [file pone.0278397.s015.docx]

**S11 Fig. Barplot proportions of the Likert Scale options by Brazil regions for even questions (Q8, Q10).**


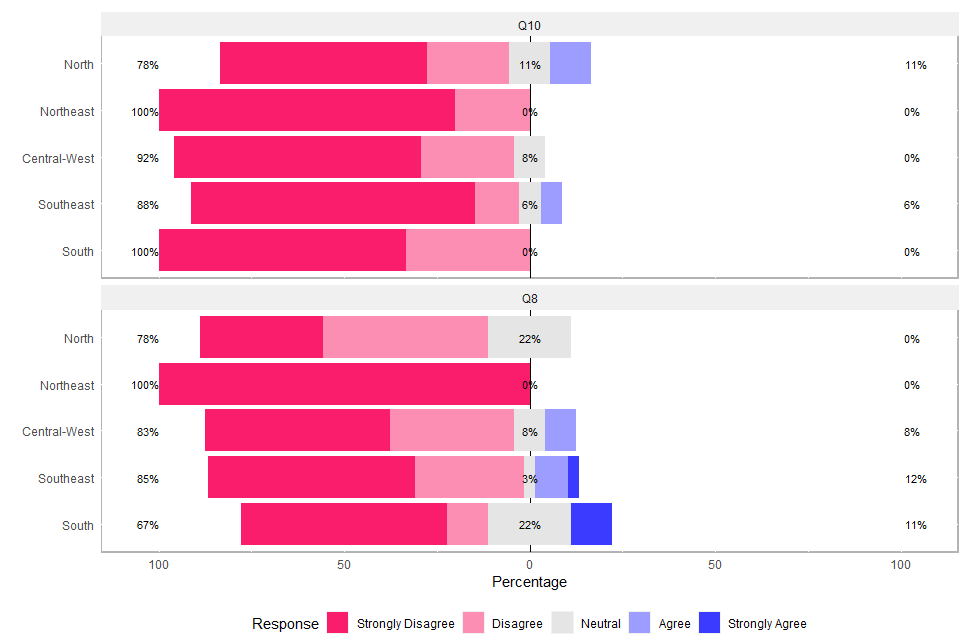

Supplement: S11 Fig — (DOCX) [file pone.0278397.s016.docx]
