# Supplementary material for: Physician preference for receiving machine learning predictive results: A cross-sectional multicentric study
Source: PLoS One. 2022 Dec 14;17(12):e0278397. doi: 10.1371/journal.pone.0278397 (PMC9749966; doi:10.1371/journal.pone.0278397)

**S6 Fig. Barplot proportions of the Likert Scale options by medical speciality for odds questions.**


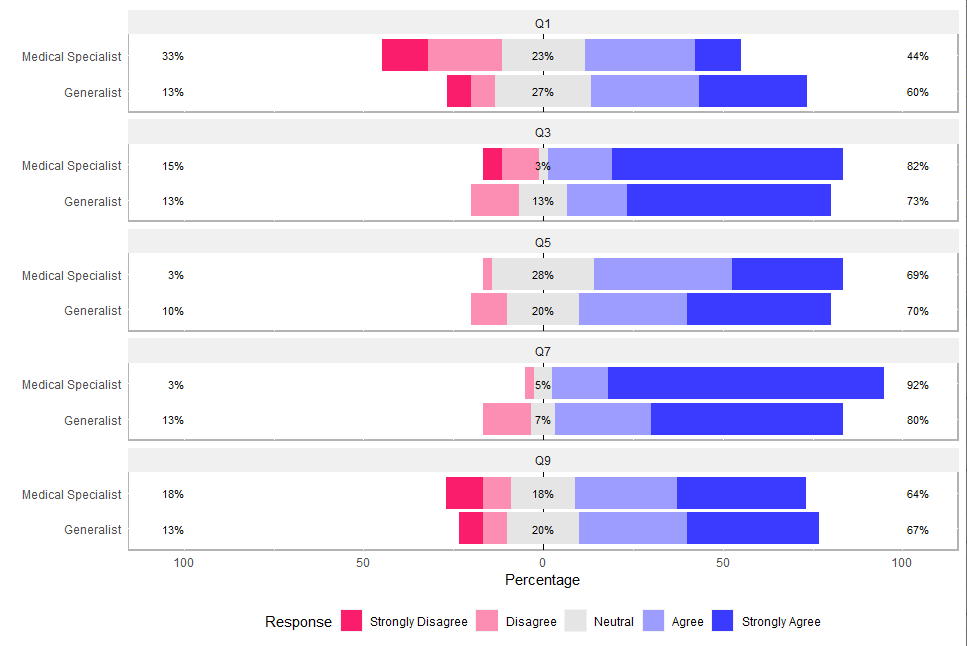

Supplement: S6 Fig — (DOCX) [file pone.0278397.s011.docx]
