# Supplementary material for: Physician preference for receiving machine learning predictive results: A cross-sectional multicentric study
Source: PLoS One. 2022 Dec 14;17(12):e0278397. doi: 10.1371/journal.pone.0278397 (PMC9749966; doi:10.1371/journal.pone.0278397)
Supplement: S12 Fig — (DOCX) [file pone.0278397.s017.docx]

**S12 Fig. Scree-plot of the eigenvalues sorted in descending order for the RandomIA questionnaire data.**

~~~~

Note: According to Figure 12, the number of eight eigenvalues was chosen because it retains the greatest amount of factor variability. This is when considering the jump in the graph that would be representing a decrease in relation to importance to the total variance, in addition to retaining eigenvalues above one unit. The proportion of the total variance explained by the eight factors is 59%
