# Supplementary material for: Physician preference for receiving machine learning predictive results: A cross-sectional multicentric study
Source: PLoS One. 2022 Dec 14;17(12):e0278397. doi: 10.1371/journal.pone.0278397 (PMC9749966; doi:10.1371/journal.pone.0278397)
Supplement: S13 Fig — (DOCX) [file pone.0278397.s018.docx]

**S13 Fig. Biplot showing the association between items plotted in the first two dimensions from the RandomIA questionnaire.**

~~
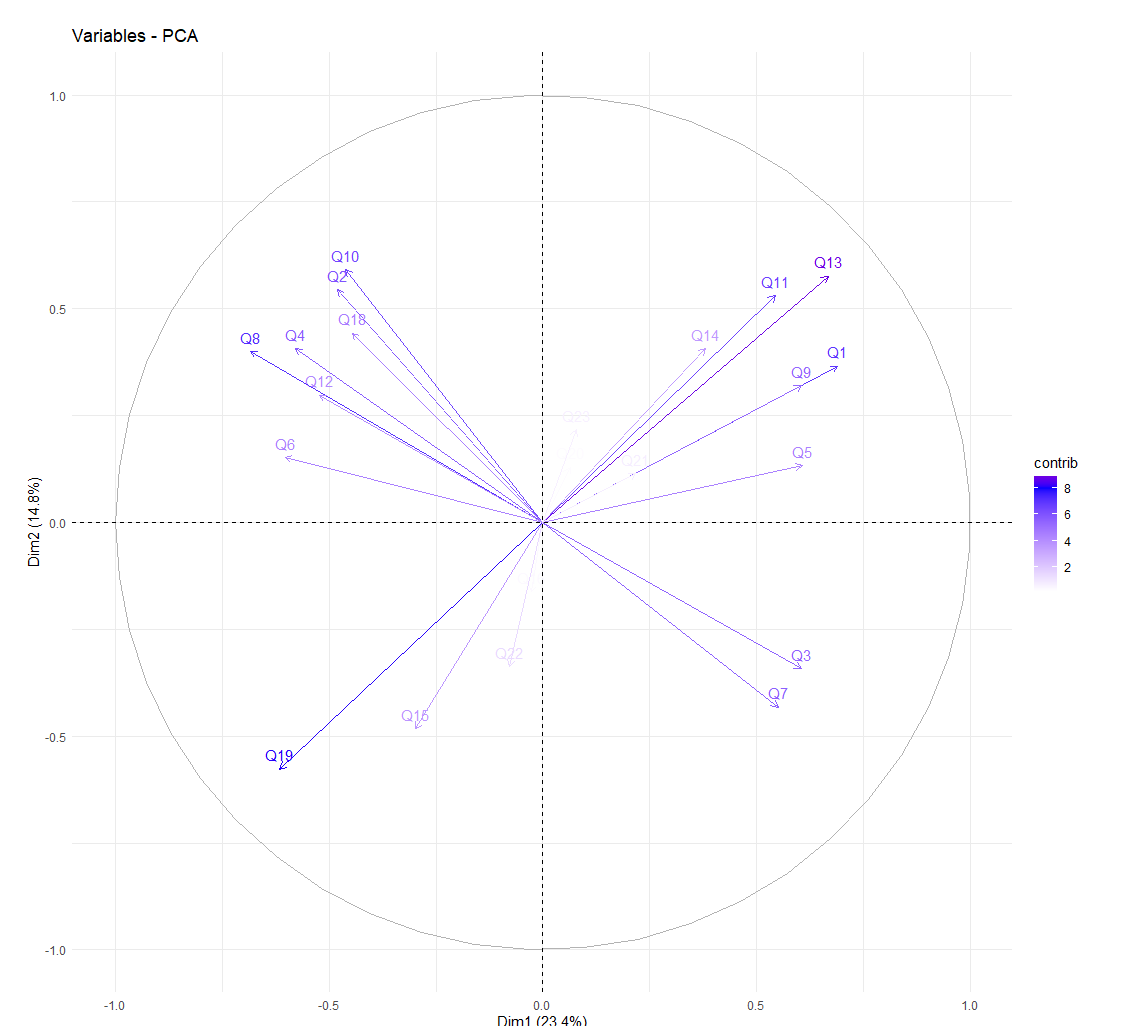
~~

Note: Questions Q14, Q11, Q13, Q9, Q1 and Q5 present similar behavior in the first two dimensions. Questions Q19, Q15 and Q22 have factor loads opposite to those of the first quadrant of the biplot. Q8, Q6, Q12, Q4, Q18, Q2, Q10 have similar behavior, while Q3 and Q7 have a similar behavior but opposite to the questions in quadrant 2 of the biplot.
