# Supplementary material for: Physician preference for receiving machine learning predictive results: A cross-sectional multicentric study
Source: PLoS One. 2022 Dec 14;17(12):e0278397. doi: 10.1371/journal.pone.0278397 (PMC9749966; doi:10.1371/journal.pone.0278397)
Supplement: S14 Fig — (DOCX) [file pone.0278397.s019.docx]

**S14 Fig. Biplot showing the dispersion of doctors in relation to the questions in the first two dimensions, based on the RandomIA questionnaire.**


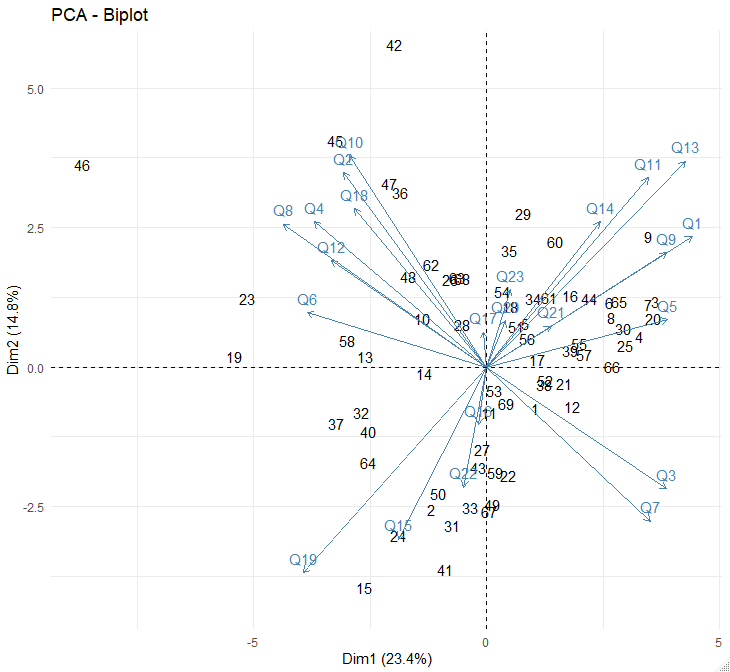


Note: Quadrant 1, linked to questions Q5, Q1, Q9, Q13, Q11, Q14, Q23, Q20, is associated with a higher concentration of participants.
