# Supplementary material for: Physician preference for receiving machine learning predictive results: A cross-sectional multicentric study
Source: PLoS One. 2022 Dec 14;17(12):e0278397. doi: 10.1371/journal.pone.0278397 (PMC9749966; doi:10.1371/journal.pone.0278397)
Supplement: S15 Fig — (DOCX) [file pone.0278397.s020.docx]

**S15 Fig. Biplot showing association of the questions in the first two dimensions from the RandomIA questionnaire by Brazilian regions.**


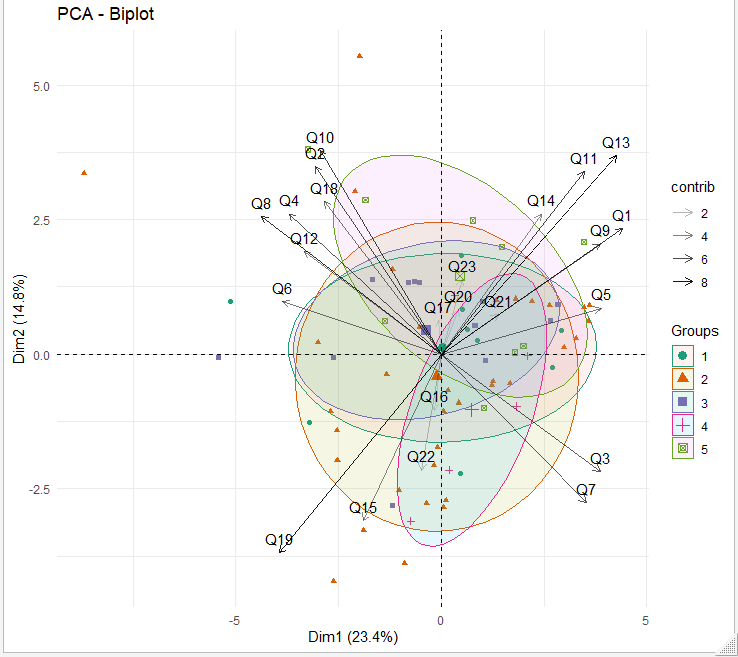


Note: Curve representing Regions 3, 4 and 5, referring to the center west, northeast, north, respectively, are ellipsoid. This suggests that the respondents from these regions have a more similar behavior compared to the responding physicians from regions 2 (southeast) and south (1).
